# Recent advances in B-cell epitope prediction methods

**DOI:** 10.1186/1745-7580-6-S2-S2

**Published:** 2010-11-03

**Authors:** Yasser EL-Manzalawy, Vasant Honavar

**Affiliations:** 1 Department of Systems and Computer Engineering, Al-Azhar University, Egypt; 2 Department of Computer Science, Artificial Intelligence Research Laboratory, Center for Computational Intelligence, Learning, and Discovery, Iowa State University, USA

## Abstract

Identification of epitopes that invoke strong responses from B-cells is one of the key steps in designing effective vaccines against pathogens. Because experimental determination of epitopes is expensive in terms of cost, time, and effort involved, there is an urgent need for computational methods for reliable identification of B-cell epitopes. Although several computational tools for predicting B-cell epitopes have become available in recent years, the predictive performance of existing tools remains far from ideal. We review recent advances in computational methods for B-cell epitope prediction, identify some gaps in the current state of the art, and outline some promising directions for improving the reliability of such methods.

## Review

Antigen-antibody interactions play a pivotal role in the humoral immune response. Antibodies bind to antigens at specific sites which correspond to the antigenic determinants or B-cell epitopes. Identification and characterization of B-cell epitopes in target antigens is one of the key steps in epitope-driven vaccine design, immunodiagnostic tests, and antibody production. B-cell epitopes typically belong to one of two classes: linear (continuous or sequential) epitopes or conformational (discontinuous) epitopes. Linear epitopes are short peptides that correspond to a contiguous amino acid sequence fragment of a protein [1,2]. Linear epitopes are usually identified using assays such as PEPSCAN. Consequently, current experimental methods offer little direct evidence indicating that each residue in the epitope does in fact make contact with one or more residues in the paratope (the part in the antibody that binds to the antigen) [3]. Conformational epitopes are composed of amino acids that although not contiguous in primary sequence, are brought into close proximity within the folded 3-dimensional protein structure. Most B-cell epitopes, although they are composed of short linear peptides, appear to be conformational epitopes.

Several experimental techniques are currently available for experimental mapping of B-cell epitopes [4]. However, the high cost and effort involved makes them impractical for application on a genomic scale. Computational techniques offer a fast, scalable, and cost-effective approach for predicting B-cell epitopes, for focusing experimental investigations and for improving our understanding of antigen-antibody interactions. Hence, there is a growing interest in the development of sophisticated computational tools for reliable prediction of B-cell epitopes.

Several computational methods for B-cell epitope prediction have been developed in recent years (e.g., [5-14]). However, the predictive performance of current methods is far from ideal [15]. To complicate matters, immunogenicity of proteins is poorly understood [16] and whether B-cell epitopes could be deciphered as an intrinsic features of the protein remains an open question [17]. Recent studies have pointed out some of the limitations of current epitope prediction methods [6,17,18]. Hence, increasing the reliability of computational methods for B-cell epitope prediction remains a major challenge in computational vaccinology [19]. In 2007, the National Institute of Allergy and Infectious Diseases (NIAID) sponsored a workshop and a meeting of a panel of immunologists and bioinformaticians in order to assess the current state of the art in epitope prediction and to identify some areas for further research [15]. One of the key goals of the workshop was to facilitate and expedite the development of improved methods for B-cell epitope prediction. The report from the workshop recommended (among other things) developing benchmark datasets, standardizing the data formats, and identifying suitable performance metrics for comparing alternative methods.

Against this background, we review recent advances in computational methods for B-cell epitope prediction, identify some gaps in the current state of the art, and outline some promising directions for improving the reliability of such methods.

### Predicting linear B-cell epitopes

Although it is believed that the majority of B-cell epitopes are conformational epitopes [20], experimental determination of epitopes has focused primarily on the identification of linear B-cell epitopes [21]. However, even in the case of linear B-cell epitopes, antibody-antigen interactions are often conformation-dependent. The conformation-dependent nature of antigen-antibody binding complicates the problem of B-cell epitope prediction. Hence, B-cell epitope prediction is less tractable than T-cell epitope prediction [22]. In what follows, we review the major approaches for predicting linear B-cell epitopes.

#### *Propensity scale methods*

Propensity scale methods [23-26] assign a* propensity value* to each amino acid which measures the tendency of an amino acid to be part of a B-cell epitope (as compared to the background). To reduce fluctuations, the score for each target amino acid residue in a query sequence is computed as the average of the propensity values of the amino acids in a sliding window centered at the target residue. The propensity scores are then used as a basis of predicting whether a given amino acid sequence residue is likely to be part of a linear B-cell epitope. Propensity scale based methods rely on the observed correlations between specific physico-chemical properties of amino acids and the antigenic determinants in protein sequences to identify the location of the linear B-cell epitopes in the query protein sequence.

The first propensity scale method for predicting linear B-cell epitopes was introduced by Hopp and Woods [27] and utilized Levitt hydorophilicity scale [28] to assign a propensity value to each amino acid. This method is based on the assumption that antigenic determinants of protein sequences correspond typically to sequence windows that contain a large number of charged and polar residues and lack large hydrophilic residues.

Subsequently, several other propensity scales have been proposed for predicting linear B-cell epitopes. For example, Parker et al. [23], Karplus et al. [24], Pellequer et al. [29] and Emini et al. [26] have proposed propensity scale based methods that use hydrophilicity, flexibility, turns, or solvent accessibility propensity scales (respectively). PREDITOP [25], PEOPLE [30], BEPITOPE [31], and BcePred [32] predict linear B-cell epitopes based on combinations of physico-chemical properties as opposed to propensity measures that rely on individual properties.

Recently, Blythe and Flower [18] have performed an exhaustive assessment of 484 amino acid propensity scales to examine the correlation between propensity scale-based profiles and the location of linear B-cell epitopes in a dataset of 50 proteins. Their study found that even the best combinations of amino acid propensities yielded B-cell epitope predictions that were only marginally better than chance. The study concluded that the performance of propensity scale based methods reported in the literature is likely to have been overly optimistic, in part due to the small size of the datasets on which the methods had been evaluated. They suggested that more sophisticated approaches (i.e., machine learning approaches) for predicting linear B-cell epitopes need to be developed and rigorously evaluated in order to advance the state-of-the-art in linear B-cell epitope prediction.

#### *Improved propensity scale methods*

Several authors have explored methods for improving the predictive performance of propensity scale methods in predicting linear B-cell epitopes. BepiPred [11] combines the hydorophilicity scale constructed by Parker et al. [23] with a Hidden Markov Model (HMM) and demonstrates a slight but statistically significant improvement in performance over the propensity scale based methods of Parker et al. [23] and Levitt et al. [28] on a test dataset of 14 proteins and 83 epitopes. Chen et al. [8] have developed an amino acid pair (AAP) antigenicity scale that assigns to each possible pair of amino acids (i.e., dipeptides), a propensity value. The resulting AAP propensities are then used to represent each peptide sequence using 400 features. Chen et al. [8] trained and evaluated a support vector machine (SVM) classifier using this representation on a dataset of 872 unique epitopes and 872 non-epitopes. They found that the SVM classifiers trained using amino acid pair (AAP) propensity derived features outperform SVM classifiers trained using amino acid propensity derived features.

#### *Machine learning methods*

Motivated by the findings of Blythe and Flower [18] and the increasing numbers of experimentally characterized linear B-cell epitopes, several authors have explored machine learning based methods for predicting linear B-cell epitopes using amino acid sequence information. ABCPred [12] uses recurrent artificial neural networks for predicting linear B-cell epitopes and was evaluated on a dataset of 700 B-cell epitopes and 700 non-epitope peptides using 5-fold cross validation tests. Input sequence windows ranging from 10 to 20 amino acids flanking the target residue, were tested and the best performance, 66% accuracy, was obtained using a window size of 16 amino acids. Söllner and Mayer [13] represent each peptide using a set of 1487 features derived from a variety of propensity scales, neighborhood matrices, and respective probability and likelihood values. Among the machine learning methods explored, they found that the best performing method, a nearest-neighbor classifier combined with feature selection, attained an accuracy of 72% on a dataset of 1211 B-cell epitopes and 1211 non-epitopes using a 5-fold cross validation test [13]. BCPred [5] and FBCPred [10] predicts linear B-cell epitopes and flexible length linear B-cell epitopes (respectively) using support vector machine (SVM) classifiers that use string kernels [33]. COBEpro [34] uses a two-step procedure for predicting linear B-cell epitopes. In the first step, an SVM classifier is used to assign scores to fragments of the query antigen. The input of the SVM is a vector of similarities between the input fragment and all training peptide fragments. In the second step, a prediction score is associated with each residue in the query antigen based on the SVM scores for the peptide fragments. Using several benchmark datasets, COBEpro has been shown to achieve a competitive performance with other linear B-cell epitope prediction methods.

### Predicting conformational B-cell epitopes

Although more than 90% of B-cell epitopes are estimated to be conformational in nature [20], most experimental as well as computational methods focus on mapping linear B-cell epitopes. However, in the past few years, there is increasing interest in methods for predicting conformational B-cell epitopes. In what follows, we review three major approaches for predicting conformational B-cell epitopes.

#### *Sequence-based prediction methods*

Sequence-based methods in predicting conformational B-cell epitopes have the advantage that they do not require the structure of the target antigen to be known. The amino acid propensity scale methods that assign a prediction score to each residue in the antigen sequence can in principle be used to predict conformational B-cell epitopes [9]. Such methods provide a baseline for evaluation of more sophisticated conformational B-cell epitope prediction methods.

A large body of work using machine learning methods for predicting protein-protein [35,36], protein-DNA [37,38], and protein-RNA [39,40] interfaces using sequence-derived features has demonstrated the feasibility of using sequence-based classifiers in reliably identifying functionally important sites in proteins. It would be interesting to explore similar sequence-based machine learning methods for reliable prediction of conformational B-cell epitopes. The development of such B-cell epitope predictors would make it feasible to identify conformational B-cell epitopes in antigenic sequences for which no solved 3D structures are available.

#### *Structure-based prediction methods*

The most accurate experimental method for identifying conformational B-cell epitopes relies on determination of the structure of antigen-antibody complexes using X-ray crystallography [41,42]. Because the number of solved antigen-antibody complexes, or for that matter, the solved antigen structures, is small relative to the number of available antigenic sequences, there are only a small number of methods that utilize 3D structure-derived information in predicting conformational B-cell epitopes.

One of the first conformational B-cell epitope predictors is the conformational epitope predictor (CEP) [7]. Given an antigen with a known structure, CEP uses accessibility of residues and spatial distance cut-off to predict linear and conformational B-cell epitopes.

DiscoTope, a method developed by Andersen et al. [9], uses a combination of amino acid statistics, spatial context, and surface accessibility of amino acids to predict conformational B-cell epitopes. Dis coTope has been shown to outperform propensity scale methods on a dataset of 76 antigen-antibody complexes. The same study also showed that predictors that combine both sequence and structure-derived features of antigens are more accurate than those that rely on either sequence or structure derived features alone [9].

The B-cell conformational epitope predictor proposed by Rapberger et al. [43] works as follows (given the 3D structure of a query antigen): (i) Fast atomic density evaluation (FADE) [44] is applied to select an antibody among a library of 26 available antibodies showing best shape complementarity to the target antigen; (ii) FastContact algorithm [45] is used to identify the most likely interaction site between the selected antibody and the target antigen; (iii) Antigen residues that show a decrease in relative solvent accessible surface area (estimated using a probe size of 3*Å*) of at least 20% in the complex are predicted as belonging to a discontinuous epitope. This method was shown to outperform the CEP method [7] using a non-redundant dataset of 26 antigen-antibody complexes from Protein Data Bank (PDB) [46].

Ponomarenko et al. [17] introduced a benchmark data set of 62 antibody-antigen complexes extracted from PDB and used it to compare two conformational B-cell epitope prediction servers (CEP and DiscoTope ) with six publicly available web servers for protein-protein binding site prediction using various approaches: i) protein-protein docking (ClusPro [47], DOT [48] and PatchDock [49]); ii) structure-based methods applying different principals and trained on different datasets (PPI-PRED [50], PIER [51] and ProMate [52]); iii) residue conservation (ConSurf [53]). Their results suggest that docking methods outperform other methods when the top ten models and bound docking were considered. However, the overall performance was found to be relatively poor (with an average AUC no greater than 0.7) for all of the methods considered.

Ellipro, a conformational B-cell epitope predictor developed by Ponomarenko et al. [54], implements a modified version of a method that was originally introduced by Thornton et al. [55] for predicting linear B-cell epitopes. Ellipro approximates a protein surface patch by an ellipsoid. Then, a protrusion index is assigned to each residue in the patch the residues are clustered according to their protrusion index values. The resulting clusters are predicted to be part of a conformational B-cell epitope. Ponomarenko et al. [54] reported that Ellipro outperforms six other structure-based predictors of protein-protein interfaces on a dataset of 39 PDB antibody-antigen complexes.

PEPITO, a method for predicting conformational B-cell epitopes introduced by Sweredoski and Baldi [56], uses a weighted linear combination of amino acid propensity scores and half sphere exposure values [57] which encode side chain orientation and solvent accessibility of amino acid residues. An improvement in performance over DiscoTope method has been reported [56].

Rubinstein et al. [58] explored the closely related problem of discriminating the antigenic determinant of an antigen from the rest of the antigen surface. They carried out an analysis of a non-redundant dataset of 53 antigen-antibody complexes. The results of their analysis suggest that epitopes can be discriminated from the rest of the antigen surface using features such as amino acid preferences, compositions of secondary structure, geometrical shape, and evolutionary conservation.

#### *Mimotope analysis -based prediction methods*

Pizzi et al. [59] have proposed an approach that combines both experimental and computational techniques for mapping B-cell epitopes. In this approach, a phage-display library of random peptides is scanned against an antibody of interest to obtain a panel of peptides (called mimotopes) that bind to the antibody with high affinity. It is assumed that this panel of mimotopes mimics the physico-chemical properties and spatial organization of the genuine epitopes. However, the precise identification of the epitope mimicked by the set of mimotopes is not straightforward since the epitope is often discontinuous (conformational) and the epitope and mimotopes do not necessarily share a high degree of sequence similarity. Moreover, some of the mimotopes may reflect noisy biological observations and should be filtered out in the analysis. Hence, several computational methods have been proposed for localizing the panel of affinity-selected peptides on the surface of a target antigen have been proposed in literature [60-65].

In general, mimotope analysis methods available in the literature differ from each other in terms of: i) how they represent the antigen structure/sequence; ii) how they align mimotopes with the target antigen structure/sequence; iii) how they cluster the mimotopes and rank the predicted epitopes. For example, PepSurf [63,64] represents the target antigen as a surface graph, wherein the nodes denote surface residues and an edge connects two nodes if the distance between the corresponding residues is lower than a specified threshold. Each mimotope is then aligned to the surface graph using a dynamic programming algorithm in order to obtain a highest scoring path in the graph. The set of highest scoring paths that are connected to each other correspond to the predicted conformational B-cell epitope. SiteLight [60] divides the antigen surface into overlapping patches and then aligns each mimotope with each patch based on a maximal bipartite matching algorithm. Mapitope [62] extracts amino acid pairs (AAPs) from mimotopes and the most statistically significant pairs (SSPs) are identified. These are then mapped on the surface of the antigen and the most elaborate and diverse clusters are identified. These are regarded as the predicted epitope candidates.

### Current developments and promising directions

In this section, we highlight recent developments, ongoing efforts, and some promising directions for developing reliable B-cell epitope prediction methods.

#### *Predicting protective linear B-cell epitopes*

Söllner et al. [14] have recently investigated the utility of predicted antigenicity, sequence variability, and conservation of post-translational-modification motifs in predicting protective linear B-cell epitopes, i.e., linear B-cell epitopes associated with biological activity. Their analysis showed that focusing on a subset of domains in the query protein sequence (e.g., conserved regions and regions lacking post-translational modification sites) can potentially improve the predictive performance of linear B-cell epitope prediction methods. El-Manzalawy et al. [6] have recently shown that a Naive Bayes classifier trained using evolutionary information (e.g., Position specific scoring matrix (PSSM) profiles obtained using PSI-BLAST [66]) outperforms propensity scale based methods in predicting protective linear B-cell epitopes. These results suggest the possibility of improving the performance of B-cell epitope prediction methods by designing classifiers that are trained on specific subclasses of B-cell epitopes (e.g., protective or neutralizing epitopes).

#### *Hybrid and consensus predictions of B-cell epitopes*

Ensemble methods that combine the predictions of several predictors often outperform individual predictors in many biomolecular sequence and structure classification tasks [67-71]. Several strategies for combining a set of predictors, *S*, into a single consensus or meta predictor exist: (i) majority voting: the score for consensus prediction is obtained by the averaging the predicted scores of the individual predictors in *S*; (ii) weighted linear combination: the consensus prediction is obtained via a weighted sum of the predictions obtained from the predictors in *S*. The weights can be assigned based on the estimated performance of the predictors on a training dataset, or optimized to minimize the prediction error of the combined predictors on a training dataset; (iii) meta-learning: A meta-classifier is trained on a training dataset using the outputs of the predictors in *S* on each input sample as input to the classifier and the corresponding class label as the desired output of the classifier.

Recently, Söllner [72] introduced an approach for developing an ensemble of linear B-cell epitope classifiers. Initially, a large number of nearest neighbor and decision tree based classifies trained using different sets of training data features has been created. A strategy based on comparing Receiver Operating Characteristic (ROC) curves of classifiers was applied to select optimal performing classifiers. Finally, an ensemble of the optimal performing classifiers was developed based on a proposed majority voting strategy, positive unanimity voting. Simply, a positive prediction is accepted if and only if all the classifiers forming the ensemble returned a positive prediction.

#### *Improved conformational B-cell epitope prediction tools*

Antigen-antibody interactions constitute a subtype of protein-protein interactions. Therefore, the development of improved conformational B-cell epitope prediction tools may benefit from recent advances in developing protein-protein interface residue prediction methods. Hence, it would be interesting to explore the development of conformational B-cell epitope predictors that utilize or adapt sophisticated protein-protein interface predictors e.g., those that make use of sequence and structure-derived features, analyses of surface patches [51,73,74] shape descriptors [75,76] or docking [77].

#### *Immune epitope database and analysis resources*

The immune epitope database (IEDB) [78,79] is perhaps the most comprehensive database of experimentally characterized B-cell and T-cell epitopes. IEDB provides users with access to several epitope-related analysis and prediction tools including: (i) several methods for predicting linear and conformational B-cell epitopes; (ii) a tool for visualizing the predicted conformational epitopes on the 3D structure of an antigen; (iii) several tools for analyzing epitope data (e.g., computing epitope conservation and epitope population coverage). IEDB allows users to retrieve both intrinsic biochemical and extrinsic context dependent information about epitopes [78]. This makes it possible to easily assemble customized datasets (e.g., the protectivity data set [14]). Additionally, several researchers have utilized IEDB to conduct meta-analyses of pathogens of interest [80-82], thereby further enhancing the utility of IEDB in the analysis and prediction of B-cell epitopes.

#### *Critical assessment of B-cell epitope prediction methods*

Given the large number of B-cell epitope prediction methods available, there is an urgent need for systematic assessment of different methods on standard benchmark datasets [15]. In practice, it is not easy to compare different methods because of several factors: inadequate documentation of the datasets, prediction methods, or the evaluation methodology employed; the unavailability of the benchmark datasets used to evaluate the methods; the unavailability of the code that implements the method (especially in the case of predictors trained using machine learning) as opposed to a server that accepts an antigen sequence or structure as input and outputs the predicted epitopes (fair comparison of alternative machine learning methods or data representations needs to be based on the same training and test datasets); differences in data formats used for inputs and outputs of the predictors.

Rigorous comparative analyses of alternative methods are indispensible for improving our understanding of the strengths and weaknesses of different B-cell epitope prediction methods and for expediting the development of improved methods. Such critical assessment of alternative methods has proven quite valuable in other tasks e.g., protein structure prediction [83], protein-protein interaction site prediction [84].

The development of standardized data representations that would allow different prediction methods to be evaluated on standardized benchmark datasets would be extremely useful not only for comparing the methods but also for developing meta-servers combining the predictions of several prediction tools [15].

The Epitopes Toolkit (EpiT) [85] represents an attempt at standardizing the development and comparison of alternative epitope prediction methods. EpiT standardizes not only the data input and output formats for the predictors but also the encoding of the predictors themselves as serialized Java objects (model files) that can be executed within the EpiT environment.

EpiT consists of two main components: i)* model builder,* an application for building and evaluating epitope predictors and serializing these models in a binary format (model files). This application is an extension of WEKA [86], a widely used open source machine learning toolkit that includes implementations of several machine learning algorithms. WEKA provides tools for data pre-processing, classification, regression, clustering, validation, and visualization. Furthermore, WEKA provides a framework for implementing new machine learning methods and data pre-processors; ii)* predictor,* an application for applying a model to test data (e.g., set of epitopes or protein sequences).

EpiT is implemented in Java (and modulo the choice of the Java platform, platform-independent). EpiT can be freely downloaded from the project web site at http://ailab.cs.iastate.edu/epit. In addition, the project web site offers a rich resource for the developers of epitope prediction tools and for EpiT users. Some examples of the useful resources available at the EpiT project web site include:

● EpiT documentation: A tutorial and an API documentation of EpiT components that includes several examples of how to build an epitope predictor and how to build an ensemble or a consensus predictor.

● An expanding Repository of Epitope Predictors (REP): Ready to use models for predicting linear B-cell epitopes using BCPred [5], FBCPred [10], and AAP [8] methods. Other researchers can contribute new epitope predictors to this repository.

● A Repository of Epitope Datasets (RED): A repository of epitope benchmark datasets made available by the authors as well as several other publicly available datasets (in WEKA format) that can be used by EpiT users to build their own customized epitope prediction tools. Researchers can contribute additional benchmark datasets to the repository.

EpiT toolkit is available under the GNU General Public License (GPL) which allows others to freely extend or modify the software so long as the modified software is also made available under the GNU GPL.

## Conclusions

Developing improved methods for predicting B-cell epitopes requires large datasets of experimentally well-characterized B-cell epitopes, and especially, antigen-antibody complexes and protective epitopes. Special care must be exercised to ensure that the datasets used to train and evaluate the predictors are of high quality:

● Constructing Non-Redundant datasets: Redundant antibody-antigen complexes should be eliminated from the datasets of B-cell epitopes used to train and evaluate B-cell epitope predictors. Otherwise, the estimated performance of predictors can be overly optimistic due to presence of similar complexes in both the training and test datasets. Most of existing linear B-cell epitope datasets [11,13,87] consist of* unique* epitopes. However, the uniqueness of epitope sequences is not a sufficient condition for non-redundancy of the dataset because a pair of* unique* linear B-cell epitopes can share a high degree of pairwise sequence similarity. Unless additional similarity reduction steps are taken to eliminate similar epitopes from the dataset, the predictive performance of B-cell epitope predictors estimated using cross-validation on such datasets can be overly optimistic; and in some cases, lead to false conclusions regarding the performance of different predictors (or the machine learning algorithms used to train the corresponding predictors) relative to each other [10].

● Identifying Non-epitope data: In the currently available conformational B-cell epitope datasets [9,17], the antigen residues that are not part of an antibody-antigen interface in the complex are treated as “non-epitope” residues. However, because each antigen can potentially bind to multiple antibodies, it is possible that some of the “non-epitope” residues may in fact be epitope residues in the complex(s) formed by the same antigen with other antibodies. In currently available linear B-cell epitope datasets comprised of whole antigen sequences [14,88], the antigen residues that are not covered by any of the* reported* epitope are treated as “non-epitope” residues. Because the experimental data available are necessarily incomplete, some of these “non-epitope” residues might in fact be epitope residues. Training an epitope predictor using a dataset in which some epitope residues are incorrectly labeled as non-epitope residues is tantamount to training the predictor on a noisy dataset. Performance estimates of the predictors on such a dataset tend to exaggerate the number of false positives (and hence the estimates of predictor performance that are based on the numbers of false positives, true positives, false negatives, and true negatives). Some authors [5,8,10,12,13] have tried to alleviate this problem by using non-epitope residues extracted from a random sample of the protein sequences in SwissProt [89]. However, a recent study [34] has shown that the resulting datasets may yield somewhat biased performance estimates. This underscores the importance for large experimentally well-characterized datasets in this field.

The use of large, non-redundant, and experimentally well-characterized datasets can help increase the accuracy of the cross-validation based estimates of the performance of B-cell epitope predictors trained on such datasets. However, it does not necessarily inform the choice of performance measures to use for comparing different predictors (or the machine learning algorithms used to train the predictors). Accuracy, sensitivity, specificity, and correlation coefficients are widely used metrics for evaluating the performance of prediction methods [90]. None of these measures when used alone provides a complete picture of the performance of the predictor. Each of these metrics is* threshold-dependent* because it describes the performance of the predictor for a given choice of the classification threshold. Moreover, it is possible to trade off one measure (e.g., sensitivity) against another (e.g., specificity). To get a more comprehensive picture of the performance of the predictor, it is useful to consider the Receiver Operating Characteristic (ROC) curve which describes the performance of the classifier over all possible choices of the classification threshold. The ROC curve is obtained by plotting the true positive rate as a function of the false positive rate or, equivalently, sensitivity versus (1-specificity) as the discrimination threshold of the binary classifier is varied. Each point on the ROC curve corresponds to a specific choice of the classification threshold, and hence a particular choice of the tradeoff between true positive rate and false positive rate. The area under ROC curve (AUC) is a useful threshold-independent summary statistic for comparing two ROC curves. The AUC is defined as the probability that a randomly chosen positive sample will be ranked higher than a randomly chosen negative sample. An AUC = 1 corresponds to a perfect predictor, whereas an AUC = 0.5 corresponds to a predictor that classifies each input sample by randomly guessing its class label. Any predictor with a performance that is better than random will have an AUC value that lies between 0.5 and 1.0. AUC is one of the most widely used metrics for comparing the performance of different B-cell epitope predictors. It is also the recommended metric for assessing the performance of B-cell epitope predictors [15]. However, a comparison of predictors based on AUC can yield misleading conclusions when the corresponding ROC curves cross. A recent study [91] has shown that AUC has a more severe limitation: Using AUC to compare different predictors is tantamount to using different misclassification cost distributions and hence different metrics to evaluate different predictors. Because the cost of misclassifying a sample is a property of the prediction problem, and not a property of the classifier, there is a need for better metrics for fair comparison of the performance of different predictors.

## List of abbreviations used

AAP: Amino Acid Pair; AUC: Area Uunder ROC Curve; CEP: Conformational Epitope Predictor; EpiT: Epitopes Toolkit; FADE: Fast Atomic Density Evaluation; GPL: General Public License; HMM: Hidden Markov Model; IEDB: Immune Epitope Database; NIAID: National Institute of Allergy and Infectious Diseases: PDB: Protein Data Bank; PSSM: Position Specific Scoring Matrix; RED: Repository of Epitope Datasets; REP: Repository of Epitope Predictors; ROC: Receiver Operating Characteristic; SSP: Statistically Significant Pair; SVM: Support Vector Machine.

## Competing interests

The author declare no competing interests.

## Authors contributions

YE and VH conceived and wrote this review article.
